# Atomistic Doping Effects on the Ideal Strength of Graphene/Aluminum Interfaces

**DOI:** 10.3390/ma18204753

**Published:** 2025-10-16

**Authors:** Wei Wang, Can Cui, Fangfang Xia, Weiwei Xu, Tieqiang Gang, Lijie Chen

**Affiliations:** 1 School of Aerospace Engineering, Xiamen University, Xiamen 361000, China; 34720180155077@stu.xmu.edu.cn (W.W.); wwxu306@xmu.edu.cn (W.X.); 2 School of Aeronautics, Chongqing Jiaotong University, Chongqing 400074, China; 634152335@cqjtu.edu.cn (C.C.); 990202100065@cqjtu.edu.cn (F.X.)

**Keywords:** atomic doping, graphene/aluminum interface, ideal strength, density functional theory

## Abstract

Generally, atomic doping is an effective method to address the weak bonding strength of the graphene/aluminum (Gr/Al) composite interface structure caused by physical adsorption, thereby enhancing the mechanical properties of the interface structure. In this paper, the nanoscopic influence mechanisms of atomic (M, including 12 types of atoms (elements)) doping in the aluminum matrix (Al) on the ideal strength of the Gr/Al interface structures are investigated based on density functional theory. The analysis of the electronic properties of the typical interface structures reveals that doping with scandium (Sc), copper (Cu) and manganese (Mn) atoms can all improve the interface binding energy of the Gr/Al structures, but their effects on the ideal strength are different. Sc doping disrupts the symmetry of the graphene structure so as to enhance the interface binding energy, but the ideal strength of the Gr/Al structures is decreased. For Cu doping it shows good compatibility with the Al matrix and the interface binding energy is enhanced through Cu alloying with the Al matrix, while the ideal strength of the interface remains basically unchanged. As for Mn doping, it causes the charge to accumulate around the Mn atoms and a resonance peak between the d_Z_^2^ orbitals of Mn and the p_x_ orbitals of Al to form, thereby improving the ideal strength of the interface structure. This study provides valuable insights for the design of Gr/Al composites by elucidating the underlying mechanisms for enhancing interface mechanical properties.

## 1. Introduction

Aluminum matrix composites, as a promising new structural material, are widely used in aerospace engineering due to their excellent mechanical properties such as light weight, high strength and high elastic modulus [1,2,3,4,5]. Graphene (Gr), a single-atom-thick layer of carbon atoms arranged in a hexagonal lattice, possesses outstanding thermal, electrical and mechanical properties, especially its ultra-high tensile strength and Young’s modulus [6,7,8]. These remarkable characteristics make graphene an ideal reinforcement material for aluminum matrix composites [9,10,11,12]. Adding a small amount of graphene to the aluminum matrix (Al) [13,14,15,16] to prepare graphene-reinforced aluminum matrix composites [17,18,19] can effectively enhance the strength of the aluminum matrix [20,21,22,23,24,25].

Previous studies [26] show that the binding energy between graphene and the aluminum matrix is a key factor affecting the mechanical properties of the composite and the microstructure of the graphene/aluminum structure (Gr/Al) influences the binding energy and mechanical properties of the composite materials. Due to the poor wettability and weak binding between graphene and the aluminum matrix, the strength of the composite material is much lower than the theoretical expectation [27]. The binding energy of graphene/aluminum composites can be improved by introducing doped elements (M) at the interface [28] to modify the aluminum matrix. Hence, selecting M-doped atoms that exhibit good wettability and chemical affinity for both graphene and aluminum, such as copper [29,30] or nickel [31], is conducive to forming a Gr/Al structure with strong binding ability. Zhang et al. [32] synthesized Ni nanoparticle-modified composites (Ni-NPs@GNP/6061Al) based on a strategy for constructing an interlocking interface structure, which enhanced the binding energy between Gr and Al matrix and the composite material exhibit excellent strengthening effect while maintaining good ductility. He et al. [33] prepared Gr/Al composites by dispersing nano-Ni modified graphene hybrids into a 6061Al matrix. At the interface, an Al_3_Ni intermetallic compound formed, which enhanced the interfacial bonding force and thereby improved the mechanical properties of the composites. The yield strength and tensile strength were 75% and 30% higher than those of aluminum alloy, respectively. Guan et al. [34] prepared pure aluminum composites reinforced with 3 wt% Gr/Cu. Compared with the aluminum matrix, the tensile strength and hardness of the Gr-Cu/Al composite increased by 77.5% and 29.1%, respectively, primarily attributing to the synergistic effects of stress transfer and dispersion strengthening. Limited by experimental characterization methods, the influence mechanism of doped atoms on the interfacial binding ability and mechanical properties of Gr/Al composites is mostly studied from simulations at the nanoscopic level.

The first-principles calculation is one of the main methods used to explore the influence of atomic doping on the structural performance of the surface/interface at the nanoscopic level. In terms of Gr/Al structure, by using first-principles calculations, Xie et al. [35] found that doped B, N and B-N atoms in the graphene layer could increase the Gr/Al binding energy by more than 10 times, and the binding energy increased with the doping concentration, especially the co-doped of B-N atoms had the most significant effect. Liu et al. [36] employed first-principles calculations to investigate the electronic structure and binding energy of the graphene(001)/Al(111) structure. The results indicated that the binding energy of the defective graphene structure with B-doped atoms (BVG)/Al was significantly higher than that of the Gr/Al intact interface. Zhu et al. [28] investigated the effects of four alloy elements (Mg, Cu, Ti and Ni) on the stability and electronic properties of the graphene/aluminum structures. The study showed that atomic doping generally enhances the binding energy through a micro-alloying effect, although the resulting stability varies by element. Mg and Cu improve stability without causing significant lattice distortion, while Ti and Ni induce considerable graphene distortion due to strong orbital hybridization. While previous studies, including Ref. [28], primarily focused on binding energy and electronic properties, the mechanisms by which dopants influence mechanical properties have not been sufficiently explored. This work expands on prior research by examining the coupled effects of atomic doping on both stability and mechanical performance. Despite model differences, such as full-layer substitution in Ref. [28] versus single-atom doping here, the findings reinforce and extend the conclusions from a mechanical perspective: moderate orbital hybridization enhances both stability and mechanical performance, while excessive hybridization may negatively impact performance.

In the present paper, 12 common atoms (elements) M (M = B, Cr, Cu, Fe, Mg, Mn, Sc, Si, Ti, V, Zn, Zr) in aluminum alloys are selected to dope and modify the Gr/Al structure, and the electronic properties of the M-doped structure (Gr/Al-M) are analyzed based on the first-principles calculation method, The Gr/Al-M structure is stretched and simulated in different directions in the plane. The influence of atomic doping on the mechanical and electronic properties of the structure is studied, and the corresponding enhancement mechanism is explored. The related research results are of certain guiding significance for the design and mechanical property improvement in graphene-reinforced aluminum matrix composites.

## 2. Modeling and Calculation Methods

Al(111) is the low refractive index surface of Al with relatively low surface energy, which is conducive to the formation of a stable surface, and it can form a Gr/Al interface structure with the Gr(0001) surface. In this paper, the Gr/Al interface structure is constructed by combining the five-layer Al(111) surface structure and the single-layer Gr(0001) surface structure in the top-fcc model, and 2 × 2 Gr(0001) and $\sqrt{3}\times \sqrt{3}$ Al(111) is selected for lattice matching to reduce lattice distortion. For all surface/interface structures such as Gr(0001), Al(111) and Gr/Al, a vacuum layer of 15 Å is added to prevent the interaction between surface/interface structures, and the *c*-axis direction is kept fixed without optimization, while only the *a*-axis and *b*-axis parallel to the interface direction are optimized. Furthermore, when conducting the stretching simulation in this paper, the *x*-direction is defined as corresponding to the armchair direction of graphene, while the *y*-direction is consistent with its zigzag direction. The biaxial directions, respectively, correspond to the *a*-axis and *b*-axis of graphene. The stress–strain relationship during stretching of the interface structures are calculated by applying an equal amount of strain increments in the uniaxial direction or the *a,b*-biaxial directions, and the atomic positions are fixed along the tensile directions without relaxation. It should be emphasized that the present model is an idealized theoretical system constructed to elucidate the intrinsic effects of dopant atoms on interfacial properties, and the conclusions are expected to provide theoretical guidance for optimizing practical composite fabrication processes.

The calculations are implemented using the Vienna Ab Initio Simulation Package (VASP),version 5.4.4, University of Vienna, Vienna, Austria. The total energy and the geometric optimization are computed based on density functional theory (DFT) [37,38] and the projector-augmented wave (PAW) [39,40,41]. The Perdew–Burke–Ernzerhof (PBE) generalized gradient approximation (GGA) functional [42,43] is applied to describe exchange–correlation effects. The Kohn–Sham wave functions are expanded using a plane-wave basis set. Van der Waals (vdW) interactions are incorporated through the DFT-D3 correction scheme [44]. The energy cutoff is set to 550 eV, and the k-point sampling grid of 6 × 6 × 6 is used. The convergence criteria for electronic self-consistency and ionic relaxation are set to 10^−5^ eV and 10^−2^ eV/Å, respectively.

## 3. Results and Discussion

### 3.1. Analysis of Electronic Properties of Interface Structures

#### 3.1.1. Structural Stability

**Figure 1:**
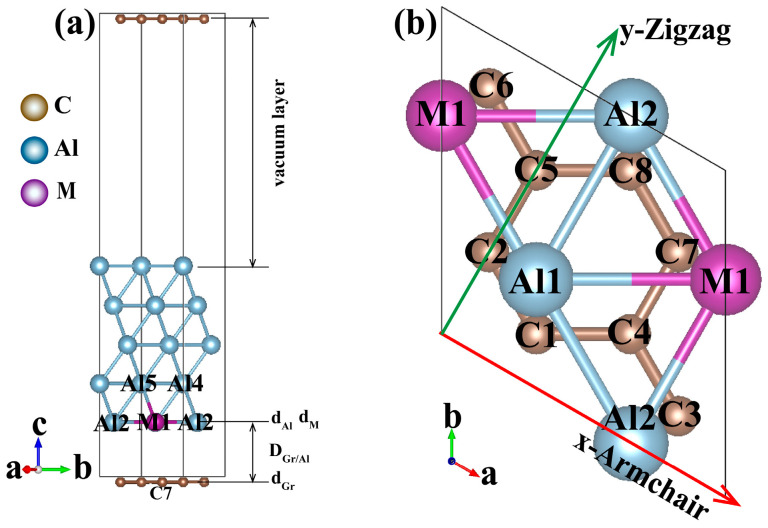
Gr/Al-M initial interface structures, (**a**) side view and (**b**) top view. Different Al and C atoms are numbered at the corresponding atomic positions.

The lattice constant of the Al crystal is 4.04 Å, that of the Al(111) surface is 2.83 Å and graphene is 2.46 Å, which are optimized. For constructing interface structures, the lattice constant difference between 2 × 2 Gr(0001) and $\sqrt{3}\times \sqrt{3}$ Al(111) is less than 2%, indicating a relatively good matching degree. One Al atom in the Al layer at the interface is substituted by the M-doped atom (M = B, Cr, Cu, Fe, Mg, Mn, Sc, Si, Ti, V, Zn, Zr), respectively. The position of the substitution atoms is shown in Figure 1a. The side view represents the Gr/Al-M interface structure containing M-doped atom. Figure 1b is the top view of the Gr/Al interface structure. For the convenience of later discussion of the deformation and stability of interface structures, some geometric parameters are defined in Figure 1. The interface spacing $D_{Gr/Al}$ are defined as the vertical distance between atoms, i.e.,

(1) $$D_{Gr/Al}=d_{Al}-d_{Gr}$$

(2) $$D_{Gr/M}=d_{M}-d_{Gr}$$

(3) $$D_{Al/M}=d_{M}-d_{Al}$$

where $d_{Al}$, $d_{Gr}$, $d_{M}$ are the vertical distances from the Al, Gr and M atoms to the reference plane with *c*-coordinate 0, respectively; $D_{Gr/Al}$ the Gr/Al-M interface spacing, i.e., the vertical distance between the graphene and Al atomic layers; $D_{Gr/M}$ the vertical distance between M-doped atom and the Gr(0001) surface; $D_{Al/M}$ the vertical distance between the M-doped atom and the Al atom.

**Figure 2:**
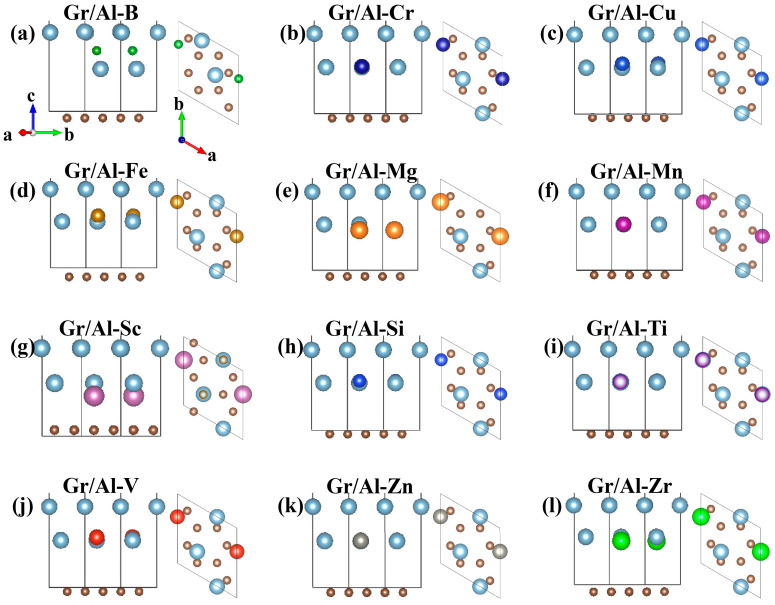
The geometrically optimized Gr/Al-M structures (**a**–**l**) are B-, Cr-, Cu-, Fe-, Mg-, Mn-, Sc-, Si-, Ti-, V-, Zn- and Zr-doped interfaces, respectively.

After optimization, the relaxed atomic configurations of the Gr/Al-M interfaces are shown in Figure 2. It is clear that the introduction of doped atoms induces significant structural deformations at the interface. When checking the positions of C atoms, it is found that slightly wrinkling appears in the graphene layer. Among the dopants, Cr, Cu, Fe, Mn, Si and V tend to migrate away from the original positions to the interior of the Al substrate, while Mg, Ti, Zn and Zr move closer to the graphene. Since the atomic radius of B and Al differs significantly, B atom preferentially occupies the interstitial site within the Al(111) matrix. Meanwhile, the absence of one Al atom reduces the Al(111) lattice constant. In contrast, Sc doping causes the Gr(0001) layer to move towards the Al(111) substrate, thereby reducing the interface distance. Combined with the performance parameters of the Gr/Al-M interface structure in Table 1, except for the unstable Gr/Al-Zn interface, all other M-doped interface structures are energetically stable.

In order to investigate the influence of M-doped atoms in the Gr/Al interface on the binding energy so as to evaluate the interface bonding strength, the variations in geometric parameters and the binding energy are computed. The Gr/Al interface structures adopt the optimal lattice constant of 4.95 Å and the initial interface spacing of 3.93 Å. After structural relaxation of Gr/Al-M structures, the lattice constants remain unchanged, while the interface spacing varies depending on the type of doped atoms. Table 1 lists the vertical distances $D_{Gr/Al}$, $D_{Gr/M}$ and $D_{Al/M}$, as well as the index of the carbon atom corresponding to the minimum $d_{Gr}$ value. The interface spacing $D_{Gr/Al}$ decreases in the following order: Gr/Al-Fe > Gr/Al-Zr > Gr/Al-Sc > Gr/Al-Mg > Gr/Al-Mn > Gr/Al-Ti > Gr/Al > Gr/Al-Cr > Gr/Al-Si > Gr/Al-Zn > Gr/Al-V > Gr/Al-Cu > Gr/Al-B. The vertical distance $D_{Gr/M}$ between the doped atom and the graphene layer follows the order of Gr/Al-B > Gr/Al-Fe > Gr/Al-Cu > Gr/Al-V > Gr/Al-Si > Gr/Al-Cr > Gr/Al-Mn > Gr/Al-Ti > Gr/Al > Gr/Al-Zr > Gr/Al-Zn > Gr/Al-Sc > Gr/Al-Mg. Similarly, the vertical spacing $D_{Al/M}$ between the dopant and Al atoms decreases in the sequence of Gr/Al-B > Gr/Al-Fe > Gr/Al-Cu > Gr/Al-V > Gr/Al-Si > Gr/Al-Cr > Gr/Al-Zn > Gr/Al-Mn > Gr/Al-Ti > Gr/Al > Gr/Al-Zr > Gr/Al-Mg > Gr/Al-Sc. These results confirm that the introduction of doped atoms induce local deformation at the interface and modifies the interface spacing, which in turn affects the interface bonding strength.

Usually, the bonding strength of interface structures can be evaluated in terms of the interface binding energy, i.e.,

(4) $$E_{b}=E_{Gr/Al-M}-\left(E_{Al/M}+E_{Gr}\right)$$

where $E_{b}$ represents the interface binding energy; $E_{Gr/Al-M}$ the total energy of the Gr/Al-M interface structure; $E_{Al/M}$ the energy of the M-doped Al substrate surface that constitutes to the interface; $E_{Gr}$ the energy of the isolated graphene surface structure from the interface.

It is generally accepted that when the interlayer spacing at the interface exceeds 3 Å and the interface binding energy is below 1 eV, the interaction between the two substances of the interface is physisorption [45]. The $E_{b}$ values of the interface binding energy after atomic doping are listed in Table 1. It suggests that the Gr/Al-M interface structures are all physical adsorption, and the influence of atomic doping on the interface binding energy is significant for Gr/Al-B, Gr/Al-Cu, Gr/Al-Fe, Gr/Al-Sc, Gr/Al-Ti and Gr/Al-Zr. It should be noted that the doping of Sc and Cu atoms can significantly improve the interface binding strength, i.e., the corresponding interface structures are of more negative binding energy.

From the aspect of the work function, describing the minimum energy value required for electrons to escape from the surface of a solid, it is one of the criteria for interface stability evaluation which can be used to further demonstrate the stability of the interface structures. In this article, the work function value (Φ) is equal to the electrostatic potential value (V_vac_, taking the maximum value) minus the Fermi level value (E_fermi_). As shown in Table 1, the work function values of the Gr/Al-M interface structure from high to low are Gr/Al-Fe > Gr/Al-Cu > Gr/Al-Zn > Gr/Al-Mn > Gr/Al-Si > Gr/Al-B > Gr/Al-Cr > Gr/Al-V > Gr/Al-Zr > Gr/Al-Sc > Gr/Al-Mg > Gr/Al-Ti > Gr/Al. This indicates that the doped atoms increase the work function of the interface structure, and electrons are less likely to escape, which means from the aspect of the work function, the M-doped interface structures are stable.

By comparing the results from the analysis of binding energy, it can be known that except for the unstable Gr/Al-Zn interface, the interface structures after doping of M atoms can all exist stably, and the doping with Sc and Cu atoms can significantly improve the interface bonding strength of the interface structures.

#### 3.1.2. Electronic Performance Analysis

Through the stability analysis of the interface structures, it is clear that after the M-doped atom replaces single Al atom, for some of the interface structures the atoms shift and the interface structure deforms. In order to have a deeper understanding of the deformation mechanism and master the electronic interaction mechanism among Al, C and M-doped atoms in the interface structures, it is necessary to calculate the electronic properties of the interface structures after atomic doping. Here, three typical modes of the interface structures after doping are selected to perform electronic properties calculations, i.e., Mn/Ti remaining relatively stable at their original positions, Cu/V migrating upward and Sc/Mg moving downward.

**Figure 3:**
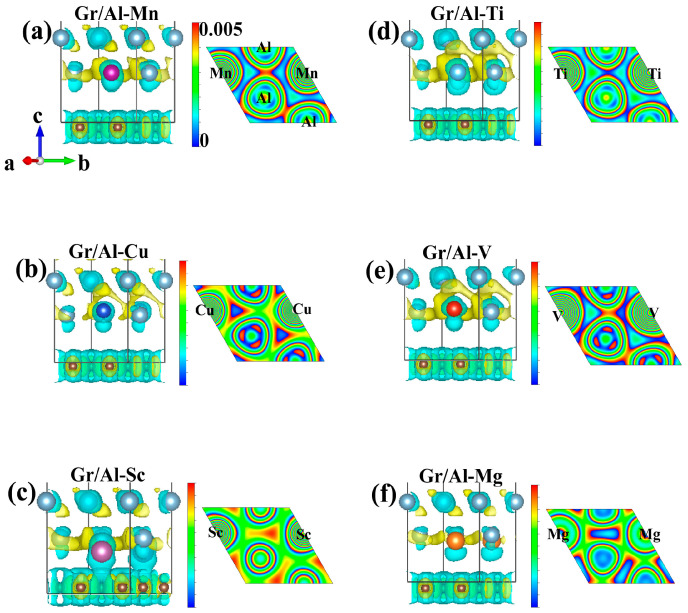
Charge density difference diagrams of the Gr/Al-M interface structures (**a**–**f**) are Mn-, Cu-, Sc-, Ti-, V- and Mg-doped, respectively. For the 2D diagram, the cross-section is based on the (100) plane where the M-doped atoms are situated. The iso-surface value is set at 0.005 electron/bohr^3^. Yellow indicates charge accumulation and blue signifies charge depletion.

The 3D side views and 2D plane views of the charge density difference for these six interface structures (Gr/Al-Mn, Gr/Al-Ti, Gr/Al-Cu, Gr/Al-V, Gr/Al-Sc and Gr/Al-Mg) are depicted, respectively, in Figure 3. It reveals that the electrons primarily accumulate around the M-doped atoms, while the Al atoms exhibit an electron depletion. Notably, the electron transfer between graphene and Al substrates is minimal. For example, the charge density difference diagram of the Gr/Al-Mn interface structure in Figure 3a reveals significant electron accumulation around the Mn atom, characterized by a localized distribution pattern. In contrast, the adjacent Al atoms show electron depletion, while the electronic distribution of graphene remains largely unaffected. The 2D slice confirms the absence of electron sharing between Mn and neighboring Al atoms, indicating weak interaction. Consequently, the enhancement of interfacial bonding strength is limited. Similar phenomena are observed in the Cu-, Ti-, V- and Mg-doped interfaces in Figure 3b–f, respectively.

In comparison, the Sc-doped interface exhibits a distinct electron interaction response, as shown in Figure 3c. A substantial amount of electron accumulation occurs around the Sc atom, accompanied by pronounced electron depletion in the surrounding Al atoms. Moreover, Sc interacts electronically with nearby carbon atoms in graphene, leading to charge redistribution at the interface. This interaction results in the formation of a delocalized electron cloud and partial electron depletion from graphene, disrupting its σ-bond between graphene layers. Such perturbations may deteriorate the intrinsic properties of graphene, including its ideal strength. These observations, in conjunction with the previous section’s results, indicate that although Sc doping can decrease the interface spacing and enhance the interface bonding strength, the intrinsic characteristics of Gr in the interface structure are disrupted. Further verification is required to ascertain whether the mechanical properties of the interface structure are decreased or not.

According to the aforementioned analysis of the charge density differences, three representative interface structures—Gr/Al-Mn, Gr/Al-Cu and Gr/Al-Sc—are selected for the presentation of their density of states (DOS) to deeply understand the electron interactions occurring at the interfaces, including electron rearrangement and electron cloud overlap among M-doped atoms, Al and C in the interface structures. These structures show strong interface bonding strength and high stability and exhibit different deformation mechanisms.

**Figure 4:**
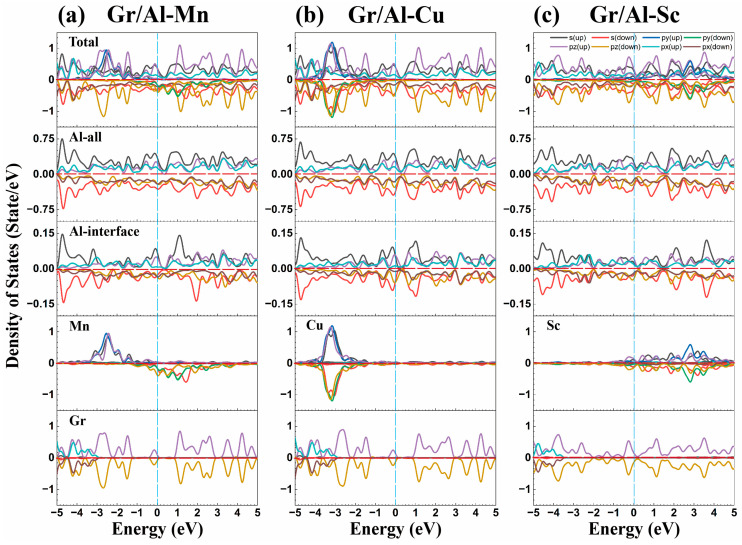
The density of states diagrams of the interface structures (**a**–**c**) are Gr/Al-Mn, Gr/Al-Cu and Gr/Al-Sc, respectively.

Figure 4 illustrates the hybridization interaction among various atomic orbitals in the Gr/Al-Mn, Gr/Al-Cu and Gr/Al-Sc interface structures. As illustrated in Figure 4, the dopant elements exert a significant influence on the graphene structure. The density of states at the Fermi level of graphene in the interface structure is non-zero, and the Dirac cone of graphene is disrupted due to interactions with aluminum and dopant atoms, resulting in a finite bandgap at the Fermi level. The hybridization between metal atoms and graphene’s π orbitals significantly enhances the metallic character of the Gr/Al structures. The peak intensities of the *d* orbitals of the doped atom and the *p* orbitals of graphene are relatively high, collectively contributing to the overall peak intensity of the interface structure, thereby enhancing the interface bonding strength. The absence of a resonance peak between these orbitals suggests that the enhancement of bonding strength is not constrained. Moreover, in Figure 4c, the Sc-doping has a pronounced effect on graphene at the interface, resulting in substantial alterations in the density of states near the Fermi level and disrupting the intrinsic electronic properties of graphene.

In summary, the charge density difference and density of states analyses for the Gr/Al-M interface structures provide qualitative insights into the electron accumulation and depletion behaviors among the M-doped atoms, Al and C atoms under different deformation modes, as well as the orbital interactions between these atoms. Among the investigated systems, the Mn-doped interface demonstrates representative electronic characteristics. Electron accumulation is observed around the Mn atom, whereas adjacent Al atoms exhibit electron depletion, with minimal influence on the graphene layer. It still allows for the bandgap of graphene to open and exhibit metallic properties. The enhancement of interface bonding strength result from the joint contribution of the *d* orbitals of Mn atoms and the *p* orbitals of graphene. The absence of a resonance peak between these orbitals indicates that the enhancement effect is relatively limited. In contrast, the electronic properties of the interface structure after Sc doping are unique. Sc doping induces electron depletion in the graphene layers and disrupts its σ-bond, thereby significantly altering the intrinsic properties of graphene.

### 3.2. Mechanical Property Analysis of Interface Structures

**Figure 5:**
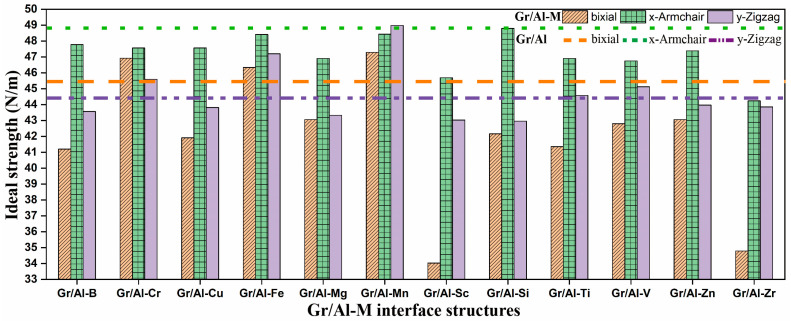
The ideal strength diagram of the Gr/Al-M interface structures when stretched along different directions, compared with the referenced dashed line of Gr/Al structures.

Building upon the structural and electronic insights obtained previously, the present section focuses on the role of atomic orbital hybridization in elucidating the mechanisms by which M doping enhances interface bonding strength. Subsequently, in-plane tensile simulations in the (100) plane along the biaxial, *x*- and *y*-directions are conducted to assess the effects of different M-doped atoms on the ideal strength of Gr/Al-M interface structures. The comparative results of ideal strength for interfaces doped with 12 different atoms are summarized in Figure 5.

Compared with the pristine Gr/Al interface structures, the Gr/Al-Cr, Gr/Al-Fe and Gr/Al-Mn interfaces exhibit higher ideal strength under biaxial tension. Under uniaxial tension along the *x*-direction, all M-doped structures experience a slight strength reduction; however, the Gr/Al-Fe, Gr/Al-Si and Gr/Al-Mn interfaces maintain strengths comparable to the undoped case. In contrast, under *y*-direction tension, the Gr/Al-Cr, Gr/Al-Fe, Gr/Al-Mn, Gr/Al-Ti and Gr/Al-V interfaces show enhanced strength, with Gr/Al-Mn achieving the most pronounced improvement. These results demonstrate that M doping can effectively improve the ideal strength of Gr/Al interfaces, with Mn providing consistent reinforcement across all three loading directions. Conversely, although Sc doping substantially strengthens interface bonding strength, it reduces strength in all directions by distrusting the intrinsic properties of graphene. Therefore, achieving simultaneous enhancement of interface bonding strength and mechanical properties requires selecting M dopants that are both compatible with the Al matrix to minimize lattice distortion and capable of preserving the intrinsic characteristics of graphene.

Table 2 summarizes the ideal strength and corresponding strain values of the Gr/Al-M interfaces under biaxial, x- and y-directions. The results show that Mn doping yields the highest overall strengthening effect, with increases of 4% under biaxial loading and 10% under *y*-direction loading, and only a negligible decrease (<0.8%) under *x*-direction loading. Moreover, the corresponding strain values are also higher, indicating improved strength without compromising ductility, an advantageous combination for practical applications. In contrast, the Gr/Al-Sc interface, despite a 1.7-fold increase in interface binding energy, suffers strength reductions of 25%, 6% and 3% under biaxial, *x*- and *y*-directions, respectively.

These results demonstrate that excessive disruption of graphene’s intrinsic symmetry, even when enhancing interface bonding strength, can be detrimental to the mechanical performance.

**Figure 6:**
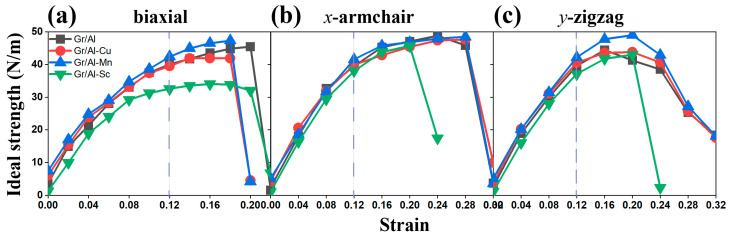
The stress–strain curves of the Gr/Al, Gr/Al-Mn, Gr/Al-Cu and Gr/Al-Sc interface structures when stretched along (**a**) biaxial, (**b**) *x*- and (**c**) *y*-directions.

To further investigate the influence of atomic doping on the mechanical properties of the interfaces, three representative structures Gr/Al-Mn, Gr/Al-Cu and Gr/Al-Sc are selected to represent cases of enhancement, negligible change and reduction in ideal strength, respectively. Their stress–strain responses (calculated using engineering strain under finite strain theory) compared with the undoped Gr/Al interface (Figure 6), show broadly similar trends, except for Gr/Al-Sc exhibiting a notably different response which highlights the dominant influence of graphene’s structural integrity. Mn doping yields a slight strength increase due to lattice distortion in the Al matrix, resulting in a solid solution strengthening effect while minimally disturbing graphene. The effect is limited with a single Mn atom, suggesting a greater potential for mono- or multilayer doping. The nearly identical strength of Cu doping interface to the undoped Gr/Al structure indicates high compatibility between Cu and the Al matrix, and structural perturbation can be ignored.

Overall, Mn doping can effectively reinforce the Gr/Al structure. For example, while maintaining ductility, the *y*-direction strength is increased by 10% and the corresponding strain by 0.04. Properly selection of transition metals with suitable atomic radii can effectively reinforce the ideal strength of the interface structures, while preserving graphene’s intrinsic structure, and multilayer doping can be considered to obtain stronger effects. 

### 3.3. Strengthening Mechanism of the Gr/Al-Mn Interface Structure

#### 3.3.1. Deformation Mechanism

M To reveal the underlying strengthening mechanisms, in this section atomic configurations, electron interactions and orbital hybridization of the Gr/Al-Mn structure during tensile deformation are further analyzed.

**Figure 7:**
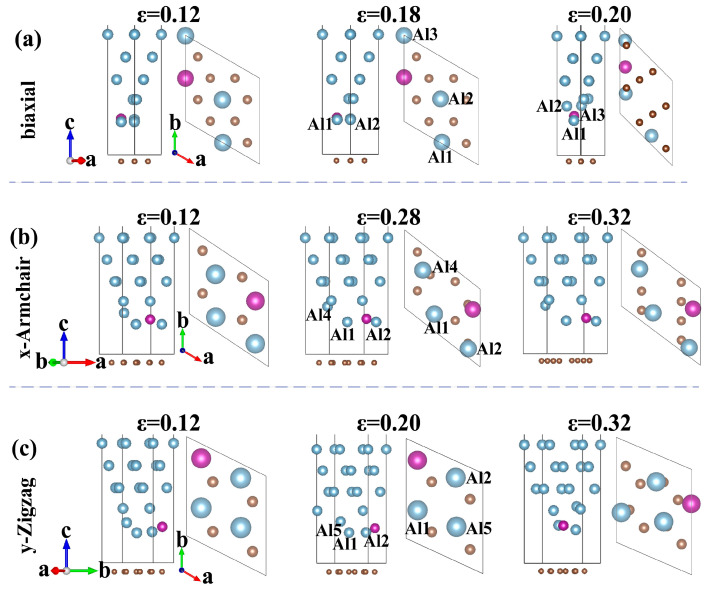
The atomic configuration diagrams of the Gr/Al-Mn interface when stretched along (**a**) biaxial, (**b**) x- and (**c**) y-directions.

Figure 7 illustrates atomic configurations of the Gr/Al-Mn interface at key tensile stages, with corresponding lattice constants and Al-Mn bond lengths summarized in Table 3. It can be found from Figure 7 and Table 3 that Mn atom migrates into the Al matrix during stretching, causing lattice distortions, especially under biaxial tension. Initially, the deformation resistance arises from the atomic displacements of Al and Mn atoms. At peak strength point, graphene undergoes displacement, while Mn atom restrains graphene, preserving critical σ and π bonds and enhancing interface stability. During the fracture stage, both Al matrix and graphene π bonds break, accompanied with Mn atom migrating deeper into Al and reinforcing the solid solution. Under *y*-direction tension, Mn acts as a “bridging” along the load path, improving Gr-Al coupling and delaying delamination. Therefore, the deformation mechanism involves initial resistance from Al, cooperative resistance from graphene and Al at peak strength aided by Mn, and final fracture characterized by broken graphene π bonds.

#### 3.3.2. Electronic Properties

**Figure 8:**
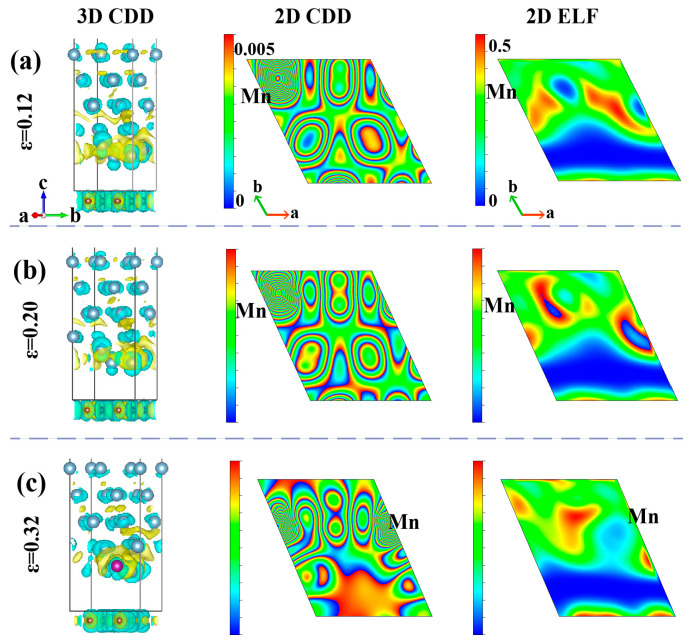
Charge density difference and local charge density of the Gr/Al-Mn interface under *y*-direction tension at strains of (**a**) 0.12, (**b**) 0.20 and (**c**) 0.32 on the (100) plane centered on the Mn. ELF values range from 0 (delocalization) to 0.5 (partial localization).

**Figure 9:**
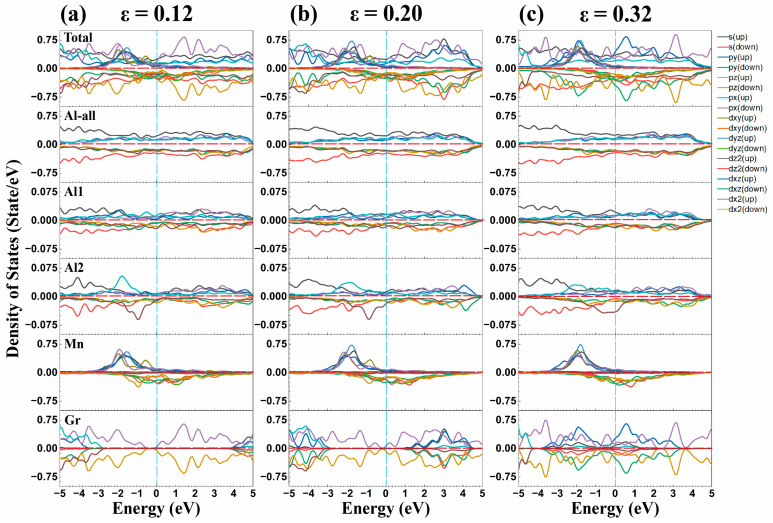
Density of states (DOS)of the Gr/Al-Mn interface under *y*-direction tension at strains of (**a**) 0.12, (**b**) 0.20 and (**c**) 0.32. Includes DOS of all Al atoms (Al-all), interface Al atoms of Al1 and Al2, Mn-doped atom (Mn) and graphene C atoms (Gr).

To further present the nanoscopic strengthening mechanism of the Gr/Al-Mn interface under *y*-direction tension, the electronic properties are systematically analyzed via charge density difference (CDD), electron localization function (ELF) and density of states (DOS), as shown in Figure 8 and Figure 9.

CDD diagrams (Figure 8) show the electron accumulation around Mn and the electron depletion near adjacent Al atoms, indicating that electron transfer f enhances Coulomb interactions and interface bonding strength. At the fracture stage, the transfer of partial electron from graphene to Mn and Al disrupts some π bonds, and no strong covalent bonds form between Mn, Al and C atoms.

ELF calculation results in Figure 8 reveal predominantly delocalization characteristics of electrons, confirming weak physical adsorption despite a 1.7-fold increase in binding energy. Mn doping strengthens Al-Mn bonding through electron delocalization, reinforcing the Al matrix and improving mechanical properties of the interface structures.

DOS results in Figure 9 show the spin-polarized *d_yz_* orbital of Mn hybridizes with the Al-*s* orbitals, while Mn-*d_z2_* and Al-*p_x_* orbitals also resonate, contributing to the interface strength. The absence of hybridization between Mn-*d* and C-*p* orbitals preserves graphene’s intrinsic properties.

In summary, the enhancement of the interface strength of Gr/Al-Mn arises primarily from (1) the maintenance of the integrity of graphene, (2) solid solution strengthening via Mn-Al electronic interactions and (3) the orbital hybridization between Mn and Al atoms. These findings can provide guidance for the design of robust metal–graphene composites.

## 4. Conclusions

In this paper, first-principles calculations are employed to investigate the ideal strength of atom-doped Gr/Al-M interface structures under in-plane tensile deformations and the atomic and electronic properties are analyzed to deeply reveal the strengthening mechanisms. The main conclusions are as follows:

(1) Sc and Cu doping significantly increase the interface binding energy, more than doubling it compared to the undoped Gr/Al interface. While Sc doping reduces interface spacing but disrupts graphene’s electronic structure. In contrast, Cu doping maintains favorable compatibility with minimal lattice distortion.

(2) Among the 12 M-doped Gr/Al-M interface structures, Mn doping leads to the most substantial enhancement in ideal strength across different loading directions. Cu doping exerts a negligible effect on the strength, while Sc doping decreases the ideal strength of the interface.

(3) The enhancement of the Gr/Al-Mn interface strength primarily attributed to the preservation of graphene integrity, the electron accumulation and solid solution strengthening induced by Mn-Al interactions, and the orbital hybridization between Mn-d_z_^2^ and Al-p_x_ orbitals. These factors synergistically contribute to an increased interfacial bonding energy and improved the mechanical performance.

## Figures and Tables

**Table 1:** Performance parameters after optimization of Gr/Al-M interface structures ($D_{Gr/Al}$, $D_{Gr/M}$ and $D_{Al/M}$ denote the interfacial vertical distances; $d_{Gr}$ the carbon atom index at the minimum; $E_{b}$ the binding energy; and Φ and E_fermi_ the work function and Fermi energy, respectively).

Models	D_Gr/Al_/Å	D_Gr/M_/Å	D	C Atoms	E_fermi_/eV	Φ/eV	E_b_/eV
Gr/Al	3.54	3.54	0.00	5, (4)	2.23	4.05	−0.41
Gr/Al-B	3.39	4.65	1.26	8	1.91	4.16	−0.31
Gr/Al-Cr	3.53	3.58	0.05	2, (3)	2.03	4.15	−0.42
Gr/Al-Cu	3.45	3.74	0.29	4, (5)	1.99	4.22	−0.91
Gr/Al-Fe	3.88	4.30	0.42	6, (4)	1.92	4.24	−0.33
Gr/Al-Mg	3.66	3.27	−0.40	2, (3)	2.19	4.06	−0.44
Gr/Al-Mn	3.55	3.56	0.01	3, (2)	2.01	4.17	−0.43
Gr/Al-Sc	3.71	2.90	−0.80	1, (8)	2.22	4.06	−1.12
Gr/Al-Si	3.51	3.66	0.15	6, (7)	2.20	4.16	−0.40
Gr/Al-Ti	3.54	3.55	0.01	2, (3)	2.08	4.05	−0.46
Gr/Al-V	3.48	3.69	0.21	3, (2)	2.04	4.13	−0.42
Gr/Al-Zn	3.49	3.51	0.02	4, (5)	1.99	4.18	−0.40
Gr/Al-Zr	3.80	3.51	−0.28	2, (3)	2.43	4.06	0.18

**Table 2:** The ideal strength and the corresponding strain of the Gr/Al-M interface structures when stretched along different directions.

Models	Ideal Strength (N/m)			Corresponding Strain		
	Biaxial	*x*	*y*	Biaxial	*x*	*y*
Gr/Al	45.44	48.81	44.41	0.20	0.24	0.16
Gr/Al-B	41.20	47.78	43.57	0.16	0.24	0.20
Gr/Al-Cr	46.92	47.57	45.60	0.18	0.28	0.26
Gr/Al-Cu	41.91	47.56	43.82	0.14	0.28	0.20
Gr/Al-Fe	46.34	48.41	47.20	0.16	0.28	0.16
Gr/Al-Mg	43.05	46.90	43.33	0.16	0.20	0.16
Gr/Al-Mn	47.29	48.43	48.98	0.18	0.28	0.20
Gr/Al-Sc	34.03	45.69	43.02	0.16	0.20	0.20
Gr/Al-Si	42.16	48.79	42.96	0.14	0.24	0.16
Gr/Al-Ti	41.36	46.91	44.57	0.12	0.20	0.20
Gr/Al-V	42.80	46.75	45.12	0.16	0.28	0.16
Gr/Al-Zn	43.06	47.39	43.97	0.16	0.20	0.16
Gr/Al-Zr	34.79	44.24	43.85	0.16	0.20	0.20

**Table 3:** Lattice constants and bond values of the Gr/Al-Mn interface structure when stretched along different directions corresponding to the strains (ε is strain; a and b the lattice constants along the corresponding crystallographic axes; γ the angle between the *a* and *b* axes; d_Al1-Mn_ and d_Al2-Mn_ the distances between the Mn atom and the first and second Al atoms, respectively).

Directions	ε	a/Å	b/Å	γ/°	d_Al1-Mn_/Å	d_Al2-Mn_/Å
biaxial	0.12	5.55	5.55	120	4.82	2.78
	0.18	5.85	5.87	120	5.10	2.89
	0.2	6.11	6.99	135	6.08	2.42
*x*	0.12	5.55	4.99	124	2.55	2.55
	0.28	6.34	5.14	128	2.58	2.64
	0.32	6.54	5.08	127	2.66	2.60
*y*	0.12	4.82	5.37	116	2.76	2.46
	0.2	4.78	5.66	115	2.89	2.44
	0.32	4.82	6.16	113	4.15	2.48

## Data Availability

The original contributions presented in this study are included in the article. Further inquiries can be directed to the corresponding author.

## References

[1] Singh J. (2016). Fabrication characteristics and tribological behavior of Al/SiC/Gr hybrid aluminum matrix composites: A review. Friction.

[2] Chen M., Fan G., Tan Z., Xiong D., Guo Q., Su Y., Zhang J., Li Z., Naito M., Zhang D. (2018). Design of an efficient flake powder metallurgy route to fabricate CNT/6061Al composites. Mater. Design.

[3] Kim W.J., Lee S.H. (2014). High-temperature deformation behavior of carbon nanotube (CNT)-reinforced aluminum composites and prediction of their high-temperature strength. Compos. Part A Appl. Sci. Manuf..

[4] Li H., Kang J., He C., Zhao N., Liang C., Li B. (2013). Mechanical properties and interfacial analysis of aluminum matrix composites reinforced by carbon nanotubes with diverse structures. Mater. Sci. Eng. A.

[5] Stein J., Lenczowski B., Fréty N., Anglaret E. (2012). Mechanical reinforcement of a high-performance aluminium alloy AA5083 with homogeneously dispersed multi-walled carbon nanotubes. Carbon.

[6] Hwang J., Yoon T., Jin S.H., Lee J., Kim T.S., Hong S.H., Jeon S. (2013). Enhanced mechanical properties of graphene/copper nanocomposites using a molecular-level mixing process. Adv. Mater..

[7] Kim W.J., Lee T.J., Han S.H. (2014). Multi-layer graphene/copper composites: Preparation using high-ratio differential speed rolling, microstructure and mechanical properties. Carbon.

[8] Lee C., Wei X., Kysar J.W., Hone J. (2008). Measurement of the elastic properties and intrinsic strength of monolayer graphene. Science.

[9] Tjong S.C. (2013). Recent progress in the development and properties of novel metal matrix nanocomposites reinforced with carbon nanotubes and graphene nanosheets. Mater. Sci. Eng. R Rep..

[10] Nieto A., Bisht A., Lahiri D., Zhang C., Agarwal A. (2016). Graphene reinforced metal and ceramic matrix composites: A review. Int. Mater. Rev..

[11] Chen Y., Zhang X., Liu E., He C., Han Y., Li Q., Nash P., Zhao N. (2016). Fabrication of three-dimensional graphene/Cu composite by in-situ CVD and its strengthening mechanism. J. Alloys Compd..

[12] Zhang X., Shi C., Liu E., Zhao N., He C. (2018). Effect of Interface Structure on the Mechanical Properties of Graphene Nanosheets Reinforced Copper Matrix Composites. ACS Appl. Mater. Interfaces.

[13] Liu J., Khan U., Coleman J., Fernandez B., Rodriguez P., Naher S., Brabazon D. (2016). Graphene oxide and graphene nanosheet reinforced aluminium matrix composites: Powder synthesis and prepared composite characteristics. Mater. Des..

[14] Hu Z., Chen F., Xu J., Nian Q., Lin D., Chen C., Zhu X., Chen Y., Zhang M. (2018). 3D printing graphene-aluminum nanocomposites. J. Alloys Compd..

[15] Bartolucci S.F., Paras J., Rafiee M.A., Rafiee J., Lee S., Kapoor D., Koratkar N. (2011). Graphene–aluminum nanocomposites. Mater. Sci. Eng. A.

[16] Yan S.J., Dai S.L., Zhang X.Y., Yang C., Hong Q.H., Chen J.Z., Lin Z.M. (2014). Investigating aluminum alloy reinforced by graphene nanoflakes. Mater. Sci. Eng. A.

[17] Rashad M., Pan F., Yu Z., Asif M., Lin H., Pan R. (2015). Investigation on microstructural, mechanical and electrochemical properties of aluminum composites reinforced with graphene nanoplatelets. Prog. Nat. Sci. Mater. Int..

[18] Wang J., Li Z., Fan G., Pan H., Chen Z., Zhang D. (2012). Reinforcement with graphene nanosheets in aluminum matrix composites. Scr. Mater..

[19] Zhao L., Lu H., Gao Z. (2014). Microstructure and Mechanical Properties of Al/Graphene Composite Produced by High-Pressure Torsion. Adv. Eng. Mater..

[20] Palei B.B., Dash T., Biswal S.K. (2022). Graphene reinforced aluminum nanocomposites: Synthesis, characterization and properties. J. Mater. Sci..

[21] Wang F., Liu H., Liu Z., Guo Z., Sun F. (2022). Microstructure analysis, tribological correlation properties and strengthening mechanism of graphene reinforced aluminum matrix composites. Sci. Rep..

[22] Yu X., Gong W., Wu H., Duan L. (2022). Mechanical and Microstructural Analysis of Exfoliated Graphite Nanoplatelets-Reinforced Aluminum Matrix Composites Synthesized via Friction Stir Processing. Arab. J. Sci. Eng..

[23] Chak V., Chattopadhyay H. (2021). Synthesis of graphene–aluminium matrix nanocomposites: Mechanical and tribological properties. Mater. Sci. Technol..

[24] Liu L.-Y., Yang Q.-S., Liu X. (2023). Microstructure design and mechanical properties of grain-gradient graphene/aluminum composites. Eng. Fract. Mech..

[25] Ju B., Yu Z., Gou H., Yang W., Chen G., Wu G. (2023). Coordinated matrix deformation induced ductility in multilayer graphene/aluminum composites. Carbon.

[26] Li M., Gao H., Liang J., Gu S., You W., Shu D., Wang J., Sun B. (2018). Microstructure evolution and properties of graphene nanoplatelets reinforced aluminum matrix composites. Mater. Charact..

[27] Shin S.E., Bae D.H. (2015). Deformation behavior of aluminum alloy matrix composites reinforced with few-layer graphene. Compos. Part A Appl. Sci. Manuf..

[28] Huang J., Wang K., Li M., Cheng Y., Lai Z., Hu J., Qu N., Liu Y., Zhou F., Zhu J. (2023). Influence of alloy atoms on the electronic structure and interfacial properties of graphene/aluminum composites: Theoretical calculation and experimental verification. Vacuum.

[29] Pu B., Mesguich D., Estournès C., Zhang X., Chevallier G., Zhao N., Laurent C. (2022). Al matrix composites reinforced by in situ synthesized graphene–Cu hybrid layers: Interface control by spark plasma sintering conditions. J. Mater. Sci..

[30] Luo Y., Huang Y., Liu J., Chen Q. (2023). Copper coated graphene reinforced aluminum composites with enhanced mechanical strength and conductivity. Vacuum.

[31] Luo Y., Huang Y., Hassan A. (2022). Fabrication and characterization of nickel-encapsulated graphene-reinforced aluminium composites. Bull. Mater. Sci..

[32] Yang L., Zhou B., Ma L., Liu G., Qian S., Xu Z., Liu E., Zhang X., He C., Zhao N. (2021). Architectured interfacial interlocking structure for enhancing mechanical properties of Al matrix composites reinforced with graphene nanosheets. Carbon.

[33] Liu G., Zhao N., Shi C., Liu E., He F., Ma L., Li Q., Li J., He C. (2017). In-situ synthesis of graphene decorated with nickel nanoparticles for fabricating reinforced 6061Al matrix composites. Mater. Sci. Eng. A.

[34] Zhao Z.Y., Guan R.G., Guan X.H., Feng Z.X., Chen H., Chen Y. (2015). Microstructures and Properties of Graphene-Cu/Al Composite Prepared by a Novel Process Through Clad Forming and Improving Wettability with Copper. Adv. Eng. Mater..

[35] Liu P., Xie J., Wang A., Ma D., Mao Z. (2020). First-principles prediction of enhancing graphene/Al interface bonding strength by graphene doping strategy. Appl. Surf. Sci..

[36] Chen Y., Liu Y., Zhou F., Chen M., Qu N., Liao M., Zhu J. (2021). The interface properties of defective graphene on aluminium: A first-principles calculation. Comput. Mater. Sci..

[37] Hohenberg P., Kohn W. (1964). Inhomogeneous Electron Gas. Phys. Rev..

[38] Kohn W., Sham L.J. (1965). Self-Consistent Equations Including Exchange and Correlation Effects. Phys. Rev..

[39] Perdew J.P., Burke K., Ernzerhof M. (1996). Generalized Gradient Approximation Made Simple. Phys. Rev. Lett..

[40] Jones R.O., Gunnarsson O. (1989). The density functional formalism, its applications and prospects. Rev. Mod. Phys..

[41] Perdew J.P., Wang Y. (2018). Erratum: Accurate and simple analytic representation of the electron-gas correlation energy [Phys. Rev. B 45, 13244 (1992)]. Phys. Rev. B..

[42] Adamska L., Lin Y., Ross A.J., Batzill M., Oleynik I.I. (2012). Atomic and electronic structure of simple metal/graphene and complex metal/graphene/metal interfaces. Phys. Rev. B..

[43] Xu L., Zheng H., Xu B., Liu G., Zhang S., Zeng H. (2023). Suppressing Nonradiative Recombination by Electron-Donating Substituents in 2D Conjugated Triphenylamine Polymers toward Efficient Perovskite Optoelectronics. Nano Lett..

[44] Huang J., Li M., Liu Y., Chen J., Lai Z., Hu J., Zhou F., Zhu J. (2023). A first-principles study on the doping stability and micromechanical properties of alloying atoms in aluminum matrix. Vacuum.

[45] Gong C., Lee G., Shan B., Vogel E.M., Wallace R.M., Cho K. (2010). First-principles study of metal–graphene interfaces. J. Appl. Phys..

